# Comparative and Functional Genomics

**DOI:** 10.1002/cfg.321

**Published:** 2003-10


					Comparative and Functional Genomics
A Special Section of Reviews from the ESF Programme on `Integrated Approaches for Functional
Genomics' First European Conference
`Functional Genomics and Disease 2003'
Local Organising Committee
V. Paces -- Chairman
M. Elleder S. Kmoch M. Macek M. Pravenec
J. Forejt J. Kopecky P. Martasek T. Ruml
J. Houstek H. Kovaoova J. Paces J. Weiser
International Programme Committee
M. Taussig -- Chairman
W. Ansorge V. De Lorenzo U. Landegren H. Schwab
P.-A. Binz M. Dunn I. Lefkovits J. Smeitink
S. Brunak J. Engels P. Legrain C. Thompson
D. Cahill A. Goffeau M. Linial M. Uhlen
P. Ceglowski A. Henriques A. Mespalu A. Valencia
J. Celis D. Higgins C. Mundy G.-J. van Ommen
M. Helmer Citterich J. Hoheisel M. Ozguc A. Varadi
A. Danchin T. Joos W. Patsch E. Vuorio
A. de Daruvar J. Kolarov H. Prydz R. Zechner
C. Dehio M. Kuiper J. Rossier
The European Science Foundation (ESF) acts as a catalyst for the development of science by bringing
together leading scientists and funding agencies to debate, plan and implement pan-European scientific
and science policy initiatives.
ESF is the European association of 76 major national funding agencies devoted to scientific research
in 29 countries. It represents all scientific disciplines: physical and engineering sciences, life and
environmental sciences, medical sciences, humanities and social sciences. The Foundation assists its
Member Organisations in two main ways. It brings scientists together in its EUROCORES (ESF
Collaborative Research Programmes), Scientific Forward Looks, Programmes, Networks, Exploratory
Workshops and European Research Conferences to work on topics of common concern including Research
Infrastructures. It also conducts the joint studies of issues of strategic importance in European science
policy.
It maintains close relations with other scientific institutions within and outside Europe. By its activities,
the ESF adds value by cooperation and coordination across national frontiers and endeavours, offers expert
scientific advice on strategic issues and provides the European forum for science.

				

